# Health risk indices and zooplankton-based assessment of a tropical rainforest river contaminated with iron, lead, cadmium, and chromium

**DOI:** 10.1038/s41598-020-72526-1

**Published:** 2020-10-09

**Authors:** Patrick Omoregie Isibor, Tunde O. Thaddeus Imoobe, Gabriel Adewunmi Dedeke, Theophilus Aanuoluwa Adagunodo, Olugbenga Samson Taiwo

**Affiliations:** 1 Department of Biological Sciences, Covenant University, PMB 1023, Ota, Ogun State Nigeria; 2 Animal and Environmental Biology, University of Benin, PMB 1154, Benin City, Nigeria; 3 Department of Physics, Covenant University, PMB 1023, Ota, Ogun State Nigeria; 4 Department of Pure and Applied Zoology, Federal University of Agriculture, PMB 2240, Abeokuta, Nigeria

**Keywords:** Zoology, Ecology, Risk factors

## Abstract

Oil exploration’s devastation on health and the environment may far outweigh its economic benefits. An oil spill occurred at Egbokodo River in Delta State, Nigeria, thereby polluting the land and water bodies. The study was therefore aimed at evaluating the impacts of iron, lead, cadmium, and chromium on the zooplankton community structure of Egbokodo River and the potential health risks. Zooplankton and surface water samples were collected to investigate the concentrations of trace metals and zooplankton abundance. The associated carcinogenic and non-carcinogenic effects of the metals in the water were analyzed. Trace metal concentrations in the surface water were determined using atomic absorption spectroscopy (Philips model PU 9100) and zooplankton samples were collected using a hydrobios plankton net (mesh size 25 µm). Total petroleum hydrocarbons (TPH) and oil and grease (OG) were determined using Agilent 7890B gas chromatography coupled to flame ionization detector (GC-FID) and volumetric analysis respectively. The trend of the abundance of zooplanktons cross the river was 18 individuals (Station A) < 100 individuals (Station B) < 155 individuals (Station C). *Cyclopoida* proved to be the most resilient to the impacts of the oil spill. On a taxa basis, the order of abundance among *Calanoida, Cyclopoida, Cladoceran*, and *Harpacticoida* was Station C > Station B > Station A, except in *Amphipoda* where Station B > Station C > Station A was observed. Iron and lead posed significant carcinogenic risks that are liable to be inflicted by the ingestion of the water. The cumulative non-carcinogenic health risk in the male was the only significant (> 1) among the age groups. Total petroleum hydrocarbons (TPH), oil and grease (OG), iron, and lead had notable impacts on the general abundance of zooplankton in the aquatic habitat. The dominance of the *Cyclopoida* in the river buttressed the impact of the oil spill which warrants a prompt remediation measure. The pollution had notable ecological impacts on the zooplankton community structure of the aquatic habitat. The adults in the nearby human populations are liable to elicit carcinogenic health challenges associated with lead and iron ingestion. The males are at risk of non-carcinogenic illnesses which are associated with the combined toxicity effects of all the metals. The study suggests that the pollution in Egbokodo River was validated by the dominance of the Cyclopoida in the aquatic habitat. The study confers bioindicator reputation on the Cyclopoida for future biomonitoring studies.

## Introduction

Zooplankton constitutes significant food items for pelagic and planktivorous fishes. These ecological roles (niche) zooplankton plays make them a major link that enhances biomagnification of trace metals across the food chain to higher trophic levels, making the concentrations reach dangerous levels in higher animals^1,2^. Zooplankton may bioaccumulate trace metals thereby poisoning the aquatic food chains through bioaccumulation at higher trophic levels. *Cladocera* and Rotifer have shown high susceptibility to trace metals in the dry season, characterized by a decline in their biodivsersity^3^. Eniola et al*.*^4^ stressed the susceptibility of zooplankton to high concentrations of trace metals^1^ and other chemical elements than phytoplankton, rapidly eliciting toxicity effects. An increase in acidity and trace elements may also harm zooplankton community structure^1,5^.

Zooplankton is of vital importance in an aquatic ecosystem because they represent a unique food source for fish and many organisms at higher trophic levels^5,6^. Nearly all fish depend on zooplankton for food during the larval stage, and some species dependence is throughout their life cycle^7^. Zooplanktons are important links in aquatic food chains converting phytoplankton or benthic plants, bacteria, fungi, and decaying organic matter into animal tissue which is incorporated into higher animals^8–10^.

Zooplankton's recovery from an oil spill may be slow if the entire population is wiped out by oil spills. Recovery is mainly through augmentation from connected water bodies. Pending recovery, the planktivorous organisms in the affected water body are deprived of the plankton component of their diet^11^. The long-lasting impacts of oil-related zooplankton mortality and the knock-on effects on other organisms in lakes and rivers warrant rigorous research on best practices that may guarantee the protection of the vital species^12,13^.

Asides being a vital link between the aquatic primary producers and secondary consumers such as phytoplankton and the nekton, zooplanktons are recyclers of water column nutrients. They are reliable bioindicators of environmental and aquatic health because of their short life-span and high sensitivity to anthropogenic environmental changes^13,14^.

Petroleum exploration and exploitation have caused devastating impacts on health and environments. Reports from various studies argue that these impacts may have far outweighed the accrued economic benefits^15,16^. Asides the exploratory processes, refining, transportation, storage, marketing, and use of petroleum products all contribute to the incidence of the oil spill in Nigeria^6,9^. During these processes, accidental spills of crude oil, petrol, lubricating oil as well as sludge and bitumen slops from tank-cleaning operations are commonly discharged on land, from where they eventually seep into water bodies through run-off and erosion of contaminated soils^7^. Extreme effects of oil spillage on water bodies in Nigeria, especially in the Niger Delta include abandonment of fish grounds, endangerment of aquatic plant and animal species, loss of good drinking water, cost of pollution clean-up, rehabilitation and relocation of people, poor human health conditions, migration of people to cause over-crowding and land pressure at the new locations, unemployment, sabotage of oil facilities, and insecurity; among other socio-cultural implications^17,15^.

An experiment conducted by O'Brien^13^ on pond oil spill bioassay indicated a sequence of sensitivity to oil toxicity among freshwater zooplankton. Fairy shrimps were the most sensitive followed by *Daphnia middentofiana*, and finally, *cyclops* which appeared to be very resistant to exposure to the oil. Lee and Nicole^18^ also found that marine calanoid copepods are more sensitive to exposure to oil than marine *Cyclopoid Copepods*.

*Calanoida* is an order of zooplankton under the subclass *Copepoda*. They include 43 families with about 2000 species of both marine and freshwater copepods. Calanoid copepods are important in many food webs, taking in energy from phytoplankton and algae and repackaging it for consumption by higher trophic level predators like birds, fish, and mammals. Many commercial fish are dependent on *Calanoida Copepoda* for diet at either their larval or adult stages^10,18^.

Pollution caused by trace metals from crude oil eliminated many rotifer species in the Bonny local government area of Rivers State and other oil exploration regions of the Niger Delta^17^. Rotifers have a higher ability to tolerate pollution-induced environmental stress than their predators and have to some extent been used as bioindicators of aquatic trace metal pollution^7,8^.

In the event of an oil spill, trace metals which are a component of crude oil, are released into the environment where they elicit persistent, bioaccumulative, and toxic attributes. Trace metals may impact on the biodiversity of aquatic biota and the well-being of human health simultaneously. Zooplankton species succession and spatial distribution result from differences in ecological response to abiotic and biotic environmental factors^10^. Although analysis of the health impacts of trace metals on adults and children through various routes of uptake has been widely reported^19–24^. Many research efforts have also been focused on pollution tolerance indices and physicochemical inferences^25,26^. There is however dearth data on the combined study of aquatic ecological and health risks of trace metals using zooplankton.

Contaminants from an oil spill in a river are liable to enter the body of humans through the consumption of fish and shellfish from the river. Such a case of ingestion is expected to elicit more severe toxicity than dermal exposure which occurs through the use of the river for washing, bathing, swimming, and other recreational uses. Furthermore, studies have shown that non-essential metals may impede the assimilation of the essential metals in the body, resulting in immunosuppression and malnutrition^15,27^.

The study was aimed at evaluating the ecological impacts of selected trace metals in Egbokodo River which was polluted with crude oil. It also seeks to evaluate the health risks associated with different routes of exposure of adults and children who use the river for various purposes. The relationship between the altered abiotic factors and the abundance of zooplankton at different locations along the river may depict vivid information about the ecological impacts of the pollution.

The study employed cancer risks and hazard indes of iron, lead, cadmium, and chromium in estimating the carcinogenic and non-carcinogenic effects respectively. These indices have proven efficient in previous studies^28^. The outcome of the study may improve the knowledge of the suitability of zooplankton as an early warning tool to the prognosis of metal exposure.

## Materials and methods

### The study area

**Figure 1:**
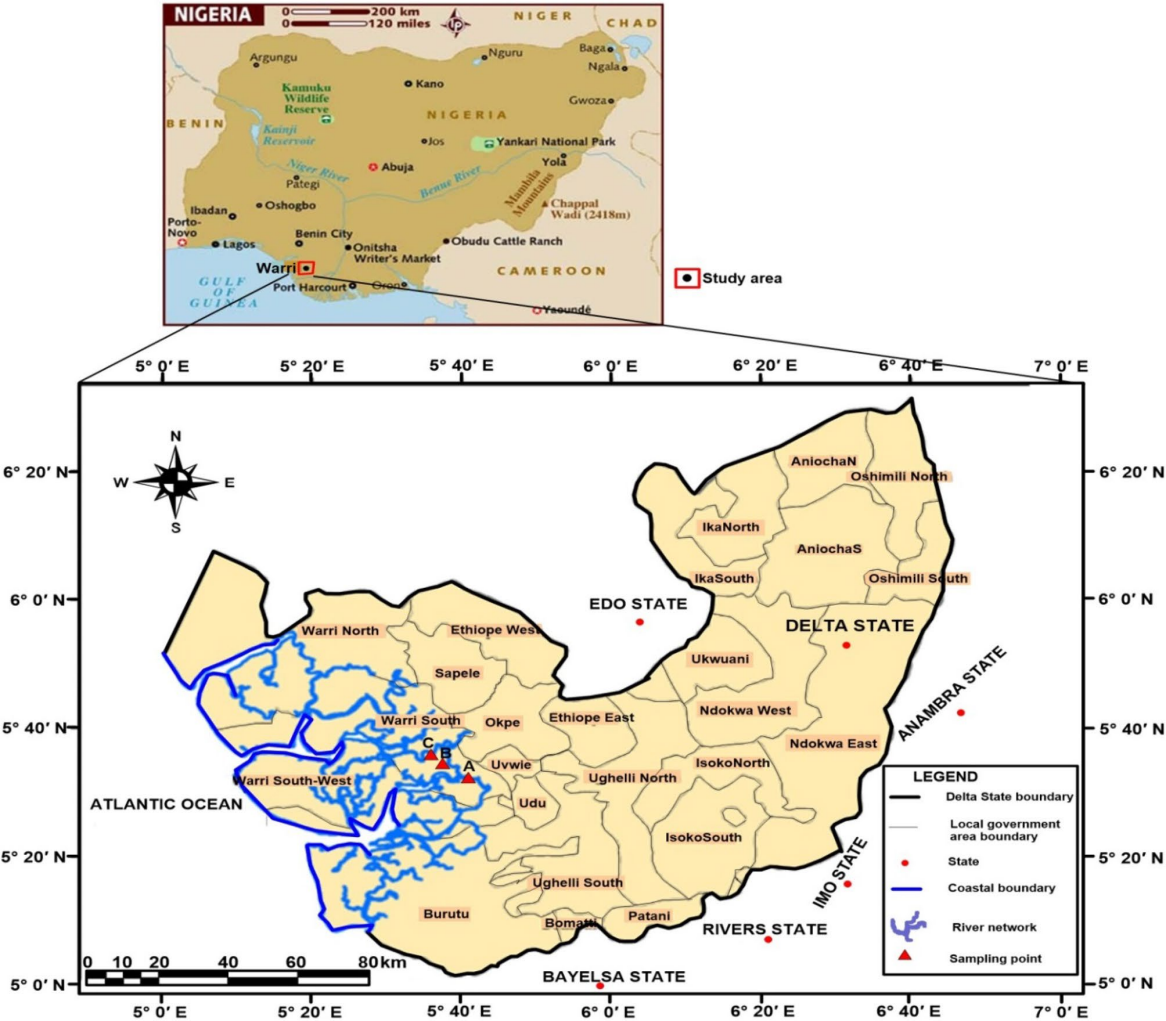
Map of the study area showing sampled stations. Map designed using QGIS software version 3.10.1 'A Coruña' (QGIS Development Team^29^). https://qgis.org/en/site/forusers/download.html#.

The study was carried out on Egbokodo River (longitude 5° 38′ and 5° 41′ and latitude 5° 36′ and 5° 33′) in Warri South Local Government Area of Delta State, Southern Nigeria (Fig. 1), between the periods of September 2008–May 2009. The river is a brackish and tidal River that serves as a source of water for drinking, washing, and fishing to the communities in the catchment area. Three (3) Stations (tagged Stations A, B, and C) were selected about 150 m apart, based on distinct anthropogenic activities. Station A was located at a vandalized oil pipeline, while Stations B and C were located downstream at points of dredging and municipal waste disposal respectively. Station A was 6.3–9.3 m in depth, Station B was 11.4–16.1, and Station C was 7.5–19.5 m during the study duration.

The study area comprises of coarse and interspersed soil with lignite and patches of laterite and sandy clay soil. The climate of the study area is typically tropical. It is characterized by the humid tropical wet and dry climate which is primarily regulated by rainfall. The wet season lasts a period of 7 months (April to October). Rainfall ranged from 15 to 91 mm during this period. The driest months are December to January; with a mean monthly rainfall of 15 mm. The bank of the river was densely shaded by a thick canopy of vegetation, dominated by mangrove plants, Nypa palm, and *Rhizophora sp.*

### Collection of samples (water and zooplankton)

Water samples were collected from the 3 stations using a 1 L sampling bottle which was pre-cleaned with the deionized water at each station. This sampling procedure was repeated monthly from September 2008 to May 2009. The samples were preserved in a cooler and transported to the laboratory where they were refrigerated at − 10 °C before the physiochemical analysis. Preservation and analysis of water samples were according to standard methods of the American Public Health Association (APHA).

Samples of zooplankton were collected at the 3 stations between 0800 and 1100 h by towing a hydrobios plankton net (mesh size 25 µm) with a speed boat at 2 knots, just below the water surface for 5 min at every station. At each station, the filtered zooplankton samples were condensed in a 25 mL plankton bottle and preserved using buffered 4% formalin. Each plankton bottle was properly labeled indicating the stations and dates of collection. This procedure was repeated for 9 months (September 2008–May 2009).

### Analysis of water

#### Determination of pH

The pH was estimated using a _P_H meter—Orion Model 290A (ASTM D 1293B) and recorded accordingly every month.

#### Measurement of temperature (°C)

A mercury-in-glass thermometer was used to measure surface water temperature. A stable initial reading was ensured by shaking it the thermometer carefully. Afterward, the thermometer was left inside the water for about 3 min till a stable reading was observed and recorded.

#### Determination of phosphate

Five (5) mL antimony molybdate was added to 40 mL of water sample was in a 50 mL measuring cylinder. Afterward, 2 mL of Ascorbic acid was added to the mixture. It was left to stand for 30 min for full colour formation^2^. The absorbance was measured with a UV–visible spectrophotometer at 680 nm.

Phosphate was then calculated thus;

1 $${\text{Phosphate}}\,({\text{mg}}/{\text{l}}) = \frac{{{\text{Y}} - {\text{C}}}}{{\text{M}}}$$

In Eq. (1) above, Y = absorbance of the sample.

C = absorbance of blank

2 $${\text{M}} = {\text{Gradient}}\frac{{({\text{B}} - {\text{A}})}}{{\text{X}}}$$

B = absorbance of standard (Eq. 2)

A = absorbance of blank

X = concentration of the standard.

#### Determination of nitrate

Nitrate was tested using the diazotization method—Alpha 419 C/ASTM D3867. 0.5 mL of (0.1% W/V) NaN_3_ was added to the water sample to remove any NO_2_ present. 3.0 mL of (2.6% W/V) NH_4_Cl solution was added. One (1) mL of (2.1% W/V) Borax solution was added. 0.5–0.6 g of spongy cadmium was added. It was then covered and shaken for some 15 to 20 min. Afterward, 7 mL of the solution was transferred to a 25 mL measuring cylinder. 1 mL of (1.0% W/V in 10% HCl) sulphanilamide reagent and was mixed by swirling. After about 3 min, 1.0 mL N-1—naphthalene diamine dihydrochloride (0.1% W/V) was added and mixed thoroughly^2^. The mark was made-up with distilled water. The blank solution was also subjected to the same treatment as the sample. After about 10–20 min, the absorbance of both the water sample and the blank solutions were measured with a UV–visible spectrophotometer at a wavelength of 543 nm.

#### Analysis of total petroleum hydrocarbons (TPH)

HP-5 capillary column coated with 5% phenyl methyl siloxane (30 m length × 0.32 mm diameter × 0.25 µm film thickness) (Agilent Technologies) was used as a stationary phase of separation of hydrocarbons from water samples. 1µL of the samples was injected in splitless mode at an injection temperature of 300 °C, and pressure of 13.74psi and a total flow of 21.364 mL/min. Purge flow to split vent was set at 15 mL/min at 0.75 min. The oven was initially programmed at 40 °C (1 min) then ramped at 12 °C/min to 300 °C for 10 min. The temperature of the flame ionization detector was regulated to 300 °C using hydrogen gas. Airflow was at 30 mL/min while nitrogen was used as makeup gas at a flow of 22 mL/min. Agilent 7890B gas chromatography coupled to flame ionization detector (GC-FID) was used for the determination of TPH at 254 nm. After calibration, water samples were analyzed and corresponding TPH concentrations were obtained^3,10^.

#### Analysis of oil and grease (OG)

One (1) L separating funnels with retort stand, 100 mL volumetric flask, glass jar, xylene, and anhydrous sodium sulfate were used in determining the concentrations of oil and grease (OG) in the water.

##### Extraction

Twenty (20) mL xylene was put in a glass jar containing a water sample. The content of the jar was shaken, poured into the separating funnel and shaken again. It was allowed for phase separation and the bottom layer xylene was drained into a 100 mL volumetric flask through a funnel with a plug of glass wool and about 2/3 full with anhydrous Na_2_SO_4_.

Another 20 mL xylene was added to the content in the separating funnel, agitated thoroughly and xylene layer was again drained into the same flask. Water was drained into a measuring cylinder and the volume was noted. Separating funnel was rinsed with 20 mL xylene into the same flask as done earlier. It was made up to mark of the extract in the 100 mL volumetric flask with pure xylene.

##### The oil and grease (OG) was calculated thus:

The concentration of oil reported as OG (mg/L)

3 $$= \frac{{{\text{Conc}}. \, \left( {{\text{mg}}/{\text{L}}\,{\text{extract}}} \right) \times {\text{DF}} \times {\text{EV}}\,{\text{(mL)}}}}{{{\text{The}}\,{\text{volume}}\,{\text{of}}\,{\text{water}}\,\,{\text{(mL)}}}}$$

In Eq. (3), DF = Dilution factor

CF = Conversion factor from absorbance to mg/L extract

EV = Extraction volume of solvent in (mL).

#### Analysis of trace metals

Ten (10) mL of water sample was put in a beaker and 2 mL concentrated nitric acid was added to the sample. The mixture was then heated to evaporation and allowed to cool afterward and then transferred into a volumetric flask. It was then allowed to stand for 24 h, after when it was centrifuged at 3000 rpm until clear. The sample was screened for suspended solids which were filtered off before further analysis. The trace metals in the mixture were then read using an atomic absorption spectrophotometer (AAS, Philips model PU 9100) at a wavelength range of 250–350 V using the ABS knob^10^.

The experimental procedures were conducted as described by Estefan et al.^30^ and modified by Jones Jr.^31^.

#### Quality control and quality assurance

##### Validation of trace metals

The precision of the AAS was validated by repeating every experimental procedure 3 times. Certified reference materials (CRM) and standard reference materials (SRM) published by the Federal Environmental Protection Agency^32^ were employed as a guide. The recovery rates ranged from 87 to 95%. The calculated relative standard deviation (SD) was < 6% (determined by Microsoft Excel, 2010), indicating high data reliability. The reference solutions used to obtain the calibration curves were prepared from analyte grade stock solutions containing 1000 mg/L of lead, iron, chromium, and cadmium^15^. The blanks and reference solutions were also analyzed using the same method that was applied to the samples. The concentrations were expressed in mg/L.

The limits of detection (LOD) and the limits of quantification (LOQ) were calculated based on the standard deviation of 20 readings obtained for the analytical blanks and the slopes of the analytical curves (LOD = 3σ/slope and LOQ = 10σ/slope). The values (mg/kg) were 0.05–0.07 µg/L for Fe, 0.07–0.123 µg/L for Pb, 0.06–0.121 µg/L for Cd, and 0.043–0.127 µg/L for Cr.

##### Validation of TPH

Total petroleum hydrocarbon readings of the GC-FID were validated by the certificate (No. TPH-R3-SET, by AccuStandard). The linearity of the calibration range was estimated to ascertain the accuracy of the equipment. The coefficient of variation of the results was validated using LODs and LOQs. The calculation of the percentage recovery for testing accuracy was also conducted. The linearity was tested between 0 and 5.6 μg/L. The LODs and LOQs were calculated from the blank and analyzed by the GC-FID. The limit of detection was calculated using triplicate readings.

### Analysis of zooplankton

Analysis of zooplankton was in the order of sorting, counting, and identification. General sorting of zooplankton samples from the 25 mL concentrate was conducted. A sub-sample of 1 mL from the collected sample was put into a hydrobios counting chamber where numbers per mL of sample were computed. Representative specimens were mounted on a glass slide in 100% glycerin pretreated with lignin pink. Relevant parts of the specimens were dissected under a binocular American Optical Corporation microscope (Model 570), using micro dissecting blades. Identification was done under an Olympus Vanox Research microscope (Model 230485) using works of literature such as Boxshall and Braide^33^, Crane^34^, Jeje and Fenando^35^, Newell, and Newell^36^, Smirnov^37^, Wickstead^38^.

#### Zooplankton diversity

The density of zooplankton was expressed as the number of organism per mL of was sample using the formula:

4 $${\text{Density}} = {\text{N}} \times {1}00/{25}\,{\text{mL}}\,{\text{(initial}}\,{\text{volume}}\,{\text{of}}\,{\text{water}}\,{\text{filtered)}}$$

N = number of zooplankton individuals per sample^3^ (Eq. 4).

The number of taxa and relative abundance was documented in detail. The zooplankton diversity was determined using indices of species diversity methods.

The indices employed include species richness, general diversity, and evenness, which were used to express the descriptive properties of the zooplankton samples at each station.

##### Margalef’s index (d)

5 $$d = \frac{S - 1}{{\ln \left( N \right)}}$$

In Eq. (5) S = total number of species, N = total number of individuals and ln is the natural logarithm (log_e_).

##### Shannon–Wienner index (H)

This index was used in calculating the general diversity of the zooplankton samples. The index was expressed as:

6 $$H = \frac{N logN - \sum ni\log ni}{N}$$

In Eq. (6) above, N = total number of individual zooplankton, n^i^ = number of individuals in the ith species, the general diversity value (H) was converted to H^i^ using the formula stated by Ogbeibu^39^ (Eq. 7):

7 $${\text{H}}^{{\text{i}}} = {2}.{3}0 \times {\text{H}}$$

##### Evenness index

The Evenness index was used to calculate the degree of evenness in the distribution of the individual zooplankton species recorded according to Ogbeibu^40^

8 $${\text{E}} = \frac{{\text{H}}}{{{\text{H}}_{\max } }}$$

In Eq. 8, H = observed diversity, H_max_ = the maximum diversity.

##### Dominance index

Simpson’s index (C) was employed to estimate the dominant species among the sampled zooplankton taxa. It was calculated thus:

9 $${\text{C}} = \sum \left( {{\text{n}}^{{\text{i}}} /{\text{N}}} \right)^{{2}}$$

n^i^ = number of individuals in the ith species, while N = total number of individuals (Eq. 9).

Using the Hutcheson t-test to calculate the significant differences according to Ogbeibu^40^, the comparison was made between two stations at a time and all were compared in pairs i.e. Station A and B, Station B and C, and Station A and C.

Hutcheson’s formula is given thus:

10 $${\text{t } = \text{ }}\frac{{{\text{H}}_{1} - {\text{H}}_{2} }}{{\sqrt {{\text{S}}^{2} {\text{H}}_{1} + {\text{S}}^{2} {\text{H}}_{2} } }}$$

In Eq. (10) above, 1 and 2 represent the two stations in comparison; H_1_ and H_2_ represent the Shannon–Weiner indices of the two stations, and S^2^H_1_ and S^2^H_2_ represent variances of Shannon–Weiner indices of the two stations.

### Health risk assessment of trace metals in water

Estimation of health risks was computed for adults (males and females separately) and children using adopted indices for referenced literature (Table 1). Data were processed for male and female adults within the age range of 40–50 years old^40^, while the data for children was a combination of male and female children within the range of 11–16 years old^41^. The adopted age brackets were representations of age frequency among the community dwellers that were observed to constantly use the river for the stated purposes.

**Table 1:** Exposure parameters and represented values adopted for estimation of human health risk of metals in Egbokodo River.

Parameters	Meaning	Adults		Reference	Children	Reference
		Male	Female			
IR	Water ingestion rate (ml/kg/day)	100	100	USDOE^41^ USEPA^42^	200	USDOE^41^
BW	Body weight (kg)	70	65	Sami et al*.*^21^ Kamunda et al*.*^20^	10	Ezemonye et al*.*^43^ USEPA^44^
CF	Conversion factor (kg/mg)	10^−6^	10^−6^	USEPA^42^	10^−6^	USEPA^44^
SA	Surface area of skin (cm^2^)	215	188	USEPA^44^	184	USEPA^41^
DAF	Dermal absorption factor	0.13	0.13	USEPA^42^	0.13	USEPA^42^
EF	Exposure frequency (day/year)	350	350	USDOE^41^	350	USDOE^41^
ED	Exposure duration (days/year)	30	30	Qu et al*.*^45^	6	Qu et al*.*^45^
AT	Average time (day)	8760	8760	Huang et al*.*^46^	2190	Huang et al*.*^46^
AF	Skin adherence factor (mg/cm^2^)	0.2	0.2	NEPAC^47^	0.2	NEPAC^47^

#### Carcinogenic risk

Considering the constant contact of the community dwellers with the river through drinking, washing, and bathing, there is a likelihood of the inhabitants having health complications at some point in their life time^2^. Health risks caused by different contaminants that enter the body through various exposure routes are generally categorized into carcinogenic and non-carcinogenic risks. Carcinogenic risks are the incremental probability of an inhabitant developing cancer in a lifetime as a result of exposure to carcinogens such as metals.

Carcinogenic risk of the river was therefore calculated according to USEPA^43^ thus:

11 $${\text{Cancer}}\,{\text{risk}}\,{\text{(CR)}} = {\text{CDI}} \times {\text{SF}}$$

The slope factor (SF) values for Fe, Cr, Pb, and Cd were adopted from Isibor et al*.*^15^, while the standard limits were adopted from USEPA^42^ for computations in Eq. (11). The modified total cancer risk was estimated according to the guidelines of USEPA^44^.

The chronic daily intake CDI for dermal (Huang et al.^47^) and ingestion (USEPA^43^) routes were calculated as stated in Eqs. 12 and 13 respectively

12 $${\text{CDI}}_{{{\text{dermal}}}} = \frac{{{\text{CW}} \times {\text{AF}} \times {\text{SA}} \times {\text{DAF}} \times {\text{CF}} \times {\text{EF}} \times {\text{ED}}}}{{{\text{BW}} \times {\text{AT}}}}$$

13 $${\text{CDI}}_{{{\text{ingestion}} - }} = \frac{{{\text{CW}} \times {\text{IR}} \times {\text{CF}} \times {\text{EF}} \times {\text{ED}}}}{{{\text{BW}} \times {\text{AT}}}}$$

CDI: chronic daily intake dose; CW: concentration of trace metal content in water (mg/L); IR: water ingestion rate (ml/kg/day); CF: conversion factor (kg/mg); EF: Exposure frequency (day/year); ED: exposure duration (years); BW: body weight (kg); AT: average time (day); AF: Skin adherence factor (mg/cm^2^); EA: exposed surface area of skin (cm^2^); DAF: dermal absorption factor (Eqs. 12, 13).

The estimated daily intake averaged in a lifetime was converted by the SF directly to the incremental risk of cancer occurrence in a lifetime^23^.

The synergistic cancer risk caused by a variety of carcinogens, which is the sum of carcinogenic risk of individual carcinogens in a common exposure route, was taken as the total CR. Usually, the value of total CR ranges from 10^−6^ to 10^−4^ (U.S. EPA^42^). Below this range was considered safe and above was declared unaccepted.

#### Non-carcinogenic risk

Trace metals are also liable to elicit non-carcinogenic health complications in the community dwellers that directly or indirectly use the river. The reference doses for the expected routes of exposure are as presented in Table 2.

**Table 2:** Reference oral and dermal doses adopted for the study.

Metals	RfD ingestion	Reference	RfD dermal	Reference
Fe	700	Li and Zhang^48^	140	Ferreira-Baptista and Miguel^50^
Pb	0.0035	Iqbal and Shah^49^	0.075	Lu et al*.*^51^
Cd	0.001	USEPA^42,44^	0.00001	Chen et al*.*^52^
Cr	0.5	USEPA^44^	0.025	USEPA^44^

*RfD* reference oral dose (mg/kg/day).

Health risk indices were calculated for the individual trace metals thus:

The hazard quotient (HQ) was calculated according to Huang et al.^47^ thus:

14 $${\text{HQ}} = {\text{CDI}}/{\text{RfD}}$$

In Eq. (14), the adopted reference doses (RfDs) for the oral (ingestion) were700, 0.001, and 3.5 × 10^−3^ mg/kg/d for Fe, Cd, and Pb respectively^48,49^, while dermal (absorption) reference doses were 40, 0.075, and 0.00001 5.25 × 10^−4^ mg/kg/d for Fe, Pb, and Cd^50–52^^.^

Estimation of the total risk posed each toxicant through a combination of the exposure routes was calculated using the total hazard quotient (∑HQ) for the male, female, and children in the population thus:

15 $$\sum {\text{HQ}} = {\text{HQ}}_{{{\text{dermal}}}} + {\text{HQ}}_{{{\text{ingestion}}}}$$

Due to possible synergistic/ antagonistic interactions among the metals analyzed^15^, the cumulative hazard quotient (HI) was estimated as the sum of the overall non-carcinogenic risk posed by individual metal (IARC^53^, NRC^54^).

It was calculated thus:

16 $${\text{HI}} = {\text{HQ}}_{{1}} + {\text{HQ}}_{{2}} + {\text{HQ}}_{{3}} $$

HQ represents hazard quotients of the individual metal analyzed (Eqs. 15, 16).

HQ or HI < 1 = unlikely to cause adverse health effects for the exposed populace, while ≥ 1 = unacceptable. The greater the values the greater the probability of the occurrence of adverse health effects in exposed individuals.

### Statistical analysis

Descriptive statistics (mean ± standard deviation) of the physicochemical properties and trace metals in water were subjected to analysis of variance (ANOVA) to test for the significant difference. Post hoc Tukey’s HSD test was used to ascertain the actual location of the significant difference. All statistical analyses were conducted at a probability level of 0.05 using SPSS 2010 version. Before ANOVA, the assumption of normality was verified using the Shapiro–Wilk test.

## Results

### The physicochemical properties of water

The temperature, total dissolved solids, turbidity, pH, dissolved oxygen, biological oxygen demand, nitrate, phosphate, sulfate, and all trace metals were within the standard limits established by FEPA^32^. The total petroleum hydrocarbons (TPH) and oil and grease (OG) at Station A were however higher than the levels at other stations and the limit established by FEPA^32^ (Table 3).

**Table 3:** Descriptive statistics of physicochemical characteristics of surface water of Egbokodo River, in comparison with the FMEnv limits.

Parameters	Station A		Station B		Station C		P-value	FEPA^32^
	Mean ± S.E	Range	Mean ± S.E	Range	Mean ± S.E	Range		
Temp (^o^C)	28.4 ± 0.567	26–30	28.5 ± 0.85	26–33	28.7 ± 0.87	25.9–32	> 0.05	–
TDS (mg/L)	1500 ± 391	72.2–3000	1669.9 ± 468.32	63.3–3820	1487.7 ± 390.73	67.5–2865	> 0.05	2000
TURB (NTU)	15.09 ± 1.04	11.14–18.90	15.45 ± 0.98	11.52–19.80	13.25 ± 0.72	10.16–17.58	> 0.05	–
pH	6.70 ± 0.09	6.03–6.99	6.63 ± 0.12	5.98–6.98	6.55 ± 0.08	6.10–6.85	> 0.05	6–9
DO (mg/L)	4.96 ± 0.34	3.50–6.40	3.51 ± 0.62	3.40–6.70	5.011 ± 0.540	2.40–7.80	> 0.05	–
BOD_5_ (mg/L)	1.27 ± 0.14	0.90–2.30	1.27 ± 0.13	0.90–2.00	1.33 ± 0.191	0.80–2.70	> 0.05	30
NO_3_ (mg/L)	0.6192 ± 0.73	0.097–1.90	1.131 ± 1.637	0.1–4.80	0.994 ± 1.379	0.046–1.980	> 0.05	20
PO_4_ (mg/L)	0.217 ± 0.23	0.0001–0.679	0.153 ± 0.068	0.0001–0.545	0.211 ± 0.074	0.0001–0.560	> 0.05	5
SO_4_ (mg/L)	37.776 ± 8.425	16.36–94.302	29.543 ± 4.225	19.35–47.10	30.956 ± 4.288	17.27–54.910	> 0.05	500
Fe (mg/L)	0.443 ± 0.100	0.045–0.860	0.488 ± 0.110	0.058–0.860	0.421 ± 0.103	0.070–0.810	> 0.05	20
Pb (mg/L)	0.073 ± 0.009	0.041–0.101	0.066 ± 0.008	0.032–0.099	0.065 ± 0.007	0.035–0.098	> 0.05	< 1
Cd (mg/L)	0.004 ± 0.001	0.0001–0.009	0.0024 ± 0.0007	0.0001–0.009	0.011 ± 0.009	0.0001–0.085	> 0.05	< 1
Cr (mg/L)	0.005 ± 0.003	0.675–0.842	0.0069 ± 0.0043	0.0001–0.040	0.0047 ± 0.0026	0.0001–0.020	> 0.05	< 1
TPH (mg/L)	2.959 ± 0.267^A^	0.64–1.510	0.883 ± 0.249^B^	0.56–1.25	0.927 ± 0.271^B^	0.66–1.425	> 0.05	2
OG (mg/L)	0.016 ± 0.002^A^	0.002–0.031	0.006 ± 0.003^B^	0.0001–0.014	0.007 ± 0.001^B^	0.002–0.013	< 0.001	10

*P < 0.001 indicates a highly significant difference. P > 0.05 indicates that there is no significant difference. Sample size (N) = 9 months. Concentrations with different superscripts are significantly different in the order of A > B

### The zooplankton community structure of Egbokodo River

The zooplankton of Egbokodo River during this study comprised 18, 100, and 155 individuals at Stations A, B, and C respectively. This makes a total of 273 individuals, belonging to 17 species and 5 taxa (Table 4). The total number of species across the stations was in the order of Station C (17) > Station B (16) > Station A (9). All 5 taxa occurred at all the stations. At Station A the order of zooplankton by percentage occurrence was *Cyclopoida* (72.2%) > *Calanoida* (11.1%) > *Amphipoda* (5.6%), *Cladocera* (5.6%), *Harpacticoida* (5.6%). At Station B the order was *Cyclopoida* (56%) > *Calanoida* (14%) > *Cladocera* (12%) > *Harpacticoida* (11%) > *Amphipoda* (7%). Station C has was *Cyclopoida* (48.4%) > *Calanoida* (29.7%) > *Cladocera* (11%) > *Harpacticoida* (7.7%) > *Amphipoda* (3.2%).

**Table 4:** Spatial distribution of zooplankton.

Species	Station A	Station B	Station C
Calanoida			
*Calanus cristatus*	1	1	3
*Cosmocalanus darwinii*	0	2	8
*Calanus brevicornis*	0	0	4
*Tropodiaptomus incognitus*	1	2	12
*Tropodiaptomus processifer*	0	4	8
*Thermodiaptomus galebi*	0	5	11
Cyclopoida			
*Thermocyclops neglectus*	4	3	16
*Thermocyclops oblongatus*	2	8	4
*Thermocyclops minutus*	0	12	5
*Cyclops bicuspidatus*	0	15	17
*Halicyclops korondiensis*	4	7	14
*Microcyclops varicans*	3	5	7
*Tropocyclops prasinus*	0	6	12
Amphipoda			
*Hyperia medusarum*	1	7	5
Cladocera			
*Chydorus eurynotus*	1	12	17
Harpacticoida			
*Harpacticus compressus*	1	11	12
Total number of individuals	18	100	155
Total number of species	9	16	17
Total number of taxa	5	5	5

#### Zooplankton biodiversity

As presented in Table 5, the species richness across the river was in the order of Station B > Station C > Station A. Result indicated that the species richness at Station B and C were significantly higher than that of Station A (p < 0.05). The Shannon–Weiner index (H) of Stations B and C was also significantly higher than that of Station A (p < 0.05). Conversely, the dominance index at Station A was significantly higher than those of Stations B and C (p < 0.05).

**Table 5:** Diversity indices of zooplankton in the river.

Indices	Station A	Station B	Station C
Species Richness Index (d)	2.768	3.25*	3.17*
Shannon–Weiner Index (H)	0.948	1.155*	1.141*
Evenness Index (E)	0.910	0.959	0.948
Dominance Index (C)	0.141*	0.075	0.083

Asterisked values are significantly higher at p < 0.05.

**Figure 2:**
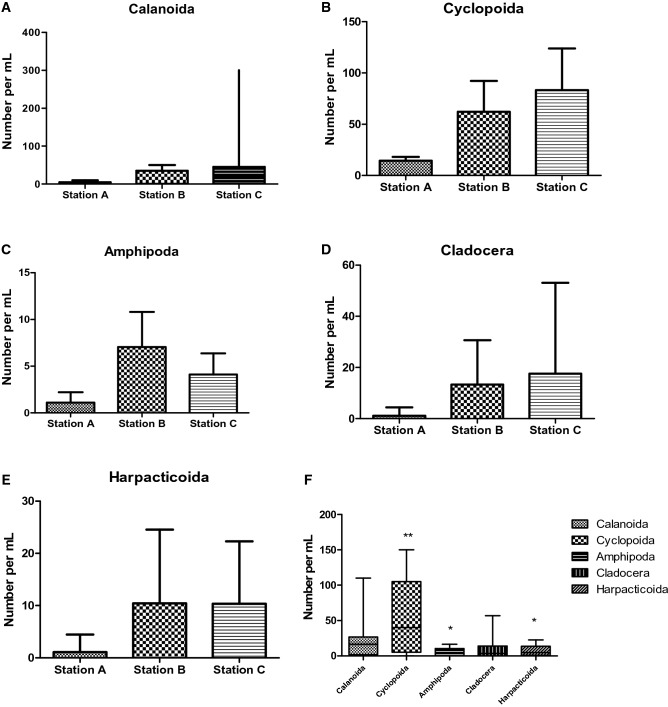
Comparative variation of zooplankton taxa. A = spatial variation of Calanoida. B = spatial variation of Cyclopoida. C = spatial variation of Amphipoda. D = Spatial variation of Cladocera. E = Spatial variation of Harpacticoida. F = Comparative abundance among zooplankton taxa of Egbokodo River. ** is significantly higher than * (p < 0.05). Sample size (N) of A–E = 6, F = 18.

Figure 2 illustrates a comparative analysis of variation within each zooplankton taxa among the stations (Fig. 2A–E) and all taxa in the entire study area (Fig. 2F). There was no significant difference (p > 0.05) in the abundance of *Calanoida* (Fig. 2A), *Cyclopoida* (Fig. 2B), and *Cladocera* (Fig. 2D) among the stations. However, the order of abundance was Station C > B > A within the taxa. A distinct order was observed among the *Amphipoda* (Fig. 2C): Station B > C > A (p > 0.05). The abundance indices of *Harpacticoida* (Fig. 2E) Stations B and C were close, which appeared outstandingly higher than the abundance at Station A but exhibited no statistical difference (p > 0.05).

A general outlook of the comparison among all the zooplankton taxa over the study period showed that the general abundance of the Cyclopoida was significantly higher (p < 0.05) than the abundances of *Amphipoda* and *Harpacticoida*, while there was no significant difference between the abundance of *Calanoida* and *Amphipoda* (p > 0.05).

Results indicated that *Cyclopoida* dominated the aquatic habitat.

#### Interrelationship between the biotic and abiotic factors

The ecological risks were inferred from the impacts of the trace metals on the abundance and distribution of the zooplankton. It is imperative to explore the correlation between the abiotic (trace metal concentrations) and biotic (zooplankton) factors. This investigation may give an insight as to whether the factors are synergistic or antagonistic in the aquatic ecosystem.

Table 6 shows that Significant negative correlations occurred between Pb and *Cyclopoida* (− 0.588), *Amphipoda* (− 0.556), and *Harpacticoida* (− 0.721). Significant negative correlation also occurred between Fe and *Calanoida* (− 0.582), *Cyclopoida* (− 0.689), *Amphipoda* (− 0.656), and *Harpacticoida* (− 0.72). A high positive correlation occurred between Cr and *Calanoida* (0.844); and *Cyclopoida* (0.582). Nitrate correlated strongly with *Cyclopoida* (0.829), and *Amphipoda* (0.993). A very high positive correlation occurred between TPH and OG. TPH also correlated negatively with the abundance of *Amphipoda* (− 0.69), while it strongly correlated negatively with the *Calanoida* (− 0.911). OG also had a strong negative correlation with the *Cyclopoida* (− 0.515), *Amphipoda* (− 0.757), and *Cladocera* (− 0.877).

**Table 6:** Correlation between abiotic factors and zooplankton species of Egbokodo River.

	Temp	pH	Cd	Pb	Fe	Cr	PO_3_	NO_3_	TPH	OG	CALA	CYCL	AMPH	CLAD	HARP
Temp	1														
pH	− 0.274	1													
Cd	− 0.342	0.082	1												
Pb	− 0.372	0.295	0.295	1											
Fe	0.034	0.198	− 0.286	**0.580**	1										
Cr	− 0.430	− 0.301	− 0.101	− 0.215	− 0.510	1									
PO_3_	**0.653**	− **0.693**	− 0.121	− 0.398	− 0.023	− 0.159	1								
NO_3_	− 0.009	0.074	− 0.209	− **0.580**	− **0.631**	0.386	− 0.227	1							
TPH	− 0.295	− 0.119	0.126	0.014	− **0.613**	**0.517**	− 0.240	**0.627**	1						
OG	− 0.262	− 0.117	0.090	− 0.062	− **0.643**	**0.521**	− 0.228	**0.692**	**0.996**	1					
CALA	− 0.486	− 0.152	− 0.043	− 0.468	− **0.582**	**0.884**	− 0.239	0.479	0.313	0.341	1				
CYCL	− 0.383	0.073	0.056	− **0.588**	− **0.689**	**0.582**	− 0.348	**0.829**	0.460	− **0.515**	**0.808**	1			
AMPH	− 0.003	0.021	− 0.220	− **0.550**	− **0.656**	0.441	− 0.197	**0.993**	− **0.696**	− **0.757**	0.484	**0.799**	1		
CLAD	− 0.240	− 0.098	0.109	0.341	− 0.345	0.353	− 0.200	0.305	− **0.911**	− **0.877**	0.020	0.068	0.396	1	
HARP	− 0.184	− 0.072	0.063	− **0.723**	− **0.721**	0.470	− 0.139	0.844	0.402	0.468	**0.715**	**0.965**	**0.809**	− 0.011	1

Emboldened values are ≥ 0.5, hence significant at p < 0.05. *OG* oil and grease, *CALA* Calanoida, *CYCL* Cylcopoida, *AMPH* Amphipoda, *CLAD* Cladocera, *HARP* Harpacticoida.

### Health risks of trace metals in Egbokodo River

#### Carcinogenic risks (CR)

**Figure 3:**
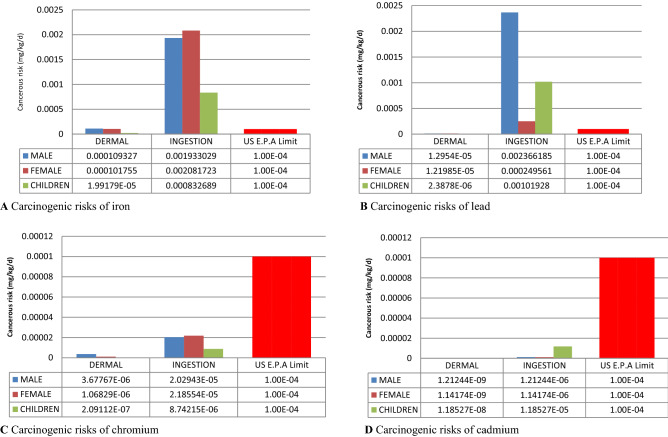
Carcinogenic health risks indices of metals.

The CR posed by the ingestion of iron in Egbokodo River to males, females, and children through ingestion exceeded the limit set by USEPA. The oral carcinogenic risks posed to the exposed population was in the order of female > male > children (Fig. 3A). Meanwhile, the dermal route of exposure to iron in the river was insignificant for all the age groups. The CRs posed by the ingestion of lead were also above the established limit in all the age groups, but contrarily in the order of male > children > female (Fig. 3B). Likewise, there was no risk associated with the ingestion of lead the population.

Conversely, chromium (Fig. 3C) and cadmium (Fig. 3D) posed no carcinogenic threat; neither through the dermal nor oral route.

#### Non-carcinogenic risks

**Figure 4:**
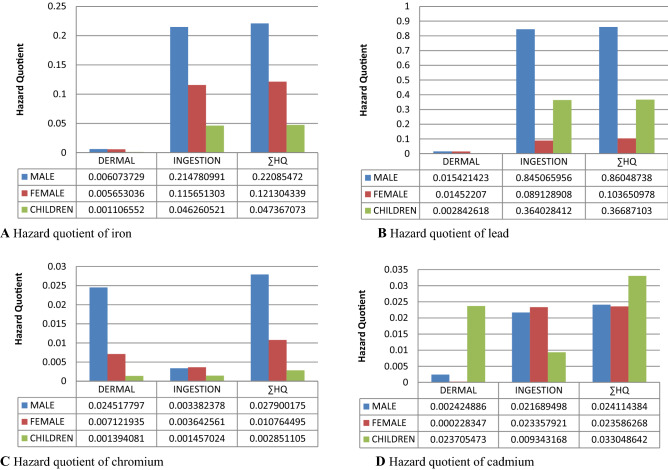
Non-carcinogenic health risks indices of metals.

The non-CR posed by all the analyzed metals were not significant in the dermal and oral exposure routes for males, females, and children alike (Fig. 4A–D).

**Figure 5:**
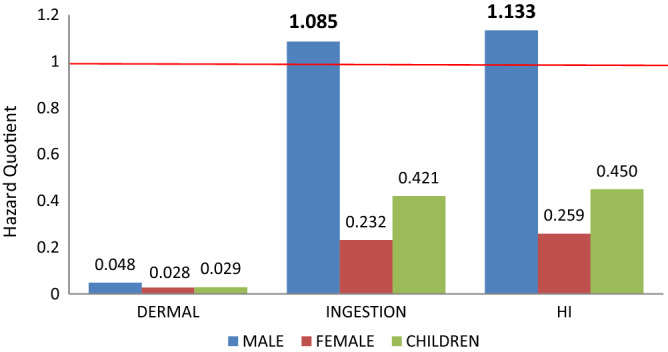
Total hazard quotients for dermal, ingestion and combined routes in males, females, and children. Emboldened and enlarged figures with a bar above the red line (> 1) represent significant hazard quotients.

The synergy of the non-carcinogenic hazard quotients among all the metals ingested and absorbed through the skin by the females and children in the population was not significant (Fig. 5). The cumulative hazard quotients (HI) which were presented as the combination of the dermal and ingestion-associated hazard indices were also not significant among the females and children. However, the HI among the males in the population was significant; marked by the high hazard quotient which exceeded the benchmark (1). The significance of the HI detected in the males was backed up by the total hazard quotient of all ingested metals which was > 1. Likewise in the cases of the females and children, there was no significant hazard associated with dermal exposure in the males of the exposed population.

The synergistic hazard indices among the metals through the dermal and oral routes were not significant among the females and children the population. The cumulative hazard quotients (HI) in the females and children were also insignificant (Fig. 5). The HI which was presented as the combined dermal and ingestion hazard indices were significant in the males, marked by the high hazard quotient which exceeded the benchmark (1). The significant HI detected in the male was due to the hazard quotients of all ingested metal, which was > 1. Likewise in females and children, there was no significant hazard associated with dermal exposure in the males of the population.

## Discussions

The pH of the aquatic habitat was fairly acidic. The pH of water is inversely proportional to the bioavailability of metals^55^. This is because, at lower pH, soluble organo-metals are formed, complex bonds are broken and metals are released as free radicals. Therefore, chances of metal uptake increase at lower pH and decrease at higher pH. This phenomenon is attributable to the bioavailability of iron in the aquatic habitat and associated potential health risks observed in this study.

The observed physicochemistry of Egbokodo River is typical of an oligotrophic freshwater habitat. Imoobe and Adeyinka^18^ earlier categorized Osse River within the same region as oligotrophic. The temperature range of 26–35 °C is characteristic of a tropical rain forest. This range also conforms to the observations of Imoobe and Adeyinka^10^.

Among the trace metals analyzed, ingested iron and lead posed significant carcinogenic threats to female > male > children; and male > children > female respectively in the population analyzed. The trend of higher carcinogenic susceptibility observed in the adults in the case of iron may due to the larger volume of blood in adults than children; being that iron is an essential component of red blood cells. The trend of male > children > female which occurred in the case of lead may be partly due to the non-essentiality of the metal, coupled with predominant occupation of fishing and boating among the male adults. While the men work to cater for their families, they remain constantly in contact with the water, thus making them the most vulnerable in the population. The children may also have ingested a significant amount of lead from the water during the frequent recreational bath which is rampant among the locals, particularly the younger ones.

Conversely, the non-carcinogenic dermal and ingestion-associated risks were not significant in all the age groups. The cumulative study of the combined non-carcinogenic hazards of all metals ingested by the males however indicated a significant risk. Previous studies have shown that individual metals may synergize to elicit accumulative toxic effects^15,56^. These were corroborated by the current results which show that among the community dwellers, the adult males that often make contact with the river are most likely to exhibit non-carcinogenic symptoms. This may be linked to the demographic characteristics of the populace, marked by the predominance of male fishermen which constitute the workforce in the population. Others are dredgers and boatmen, which are all in constant contact with the water. In this study, results showed that the adults (male and female) are at greater carcinogenic risks, while the males are at non-carcinogenic risks. These threats are liable to be elicited by lead, followed by iron in the river. This observation is consistent with that of Khalili et al*.*^57^ who observed that lead posed the greatest threat to human health among the analyzed trace metals in hair dye. Conversely, Anani and Olomukoro^56^ enlisted cadmium and chromium as the leading causes of health hazards in Ossiomo River. Furthermore, they discovered greater health risks in children than adults exposed to Pb, Cd, and Cr in Ossiomo River in Edo State which is also within southern Nigeria; a location close to the current study area.

In this study, negative strong correlations of the metals, particularly lead and iron with the abundance of the zooplankton species suggest that the metals might have impacted the biota of the aquatic habitat. This further implicates lead and iron as the main drivers of possible ecological risks, asides the associated health risks.

The levels of TPH and OG at Station A are evidence of the oil spill. More so, Station A was the actual location of the pollution. Toxicants may relapse as the toxicant might have precipitated to the bottom of the water for future re-pollution incidences^9,58–62^.

Relatively high OG at Station A, accompanied by low zooplankton abundance is attributable to the incidence of oil spill at the station. The number of zooplanktons at Station A (18 individuals) < Station B (100 individuals) < Station C (155 individuals) shows a steady increase in zooplankton abundance with distance from Station A. This further validates the station as the location of the pollution. Percentage density occurrence being *Cyclopoida* (72.2%) > *Calanoida* (11.1%) > *Amphipoda* (5.6%), *Cladocera* (5.6%), *Harpacticoida* (5.6%) at Station A, suggests that the group *Cyclopoida* thrived best at the location. As expected, *Calanoida* was outnumbered by the *Cyclopoida*^48^.

Manual dredging activities at the river may agitate the water, causing dissolution of oxygen at the air–water interphase, and increase turbidity through disturbed bottom sediment. Oxygenated and hypolimnetic condition of the river might have fostered *Calanoida*’s dominance over *Amphipoda, Cladocera,* and *Harpacticoida*. This is because though the *Calanoida* is a relatively larger zooplankton, they are capable of taking refuge in the turbidity from predators^9,63^. They also have divergent reproductive strategies which give them an edge in the event of severe anthropogenic perturbation^64–67^.

Inter-taxa comparison of abundance across the river showed that *Cyclopoida* had the highest abundance, followed by the *Calanoida* which was significantly higher than the *Cladocera*, which was in turn higher than the *Harpacticoida* and *Amphipoda*. The current observation corroborates the findings of O’Brien^13^ who demonstrated high toxicity of crude oil on fairy shrimps (*Branchionecta paladosa*), followed by *Daphnia midendofiana*, while *Heteroscope septetrionalis* exhibited appreciable resistance. O’Brien^13^ observed that *Cyclopod copepods* were the only species that survived the oil contamination at the end of 21 days. Ikhuoriah et al*.*^1^ reported the dominance of *Thermocyclops neglectus* over other species in Ossiomo River, Edo State, Nigeria.

Diversity analysis further indicated that the species richness (d) and Shannon–Weiner index (H) at Station B, followed by Station C were both significantly higher than the values at station A (p < 0.05). Conversely, the significantly higher dominance index (I) observed at Station A than other stations (p < 0.05) further indicates that Station A was dominated by a particular species, while nearly inhabitable to the others. Linking the percentage density at Station A with the diversity indices suggests that Cyclopoida were the dominant species across the study area, particularly at the location of the oil spill.

Rotifer species *Conochilus dossuarius* and *Synchaeta longipes* that are typical of oligotrophic to mesotrophic systems was absent in the river. Worse still, *Keratella tropica, Keratella quadrata, Brachionus angularis, Trichocerca pusilla, Filinia longiseta, Pompholyx sulcata* and *Proales sp.* that are usually most predominant species in West Africa freshwater ecosystems which are indicator species of high trophic levels^17^ were also not recorded in the river^15,52^. The absence of these vital species in the river indicates a poor trophic status which is evident in the spatiotemporal profiles of nitrates and phosphates.

The occurrence of *Calanoida* species (mainly *Tropodiaptomus incognitus, T. processifer* and *Thermodiaptomus galebi*) and Cyclopoida (*Thermocyclops neglectus, Microcyclops varicans* and *Halicyclops korondiensis*) corroborates the work of Imoobe and Adeyinka^15^ and was supported by Isibor^10^ who inferred an oligotrophic status from the concurrent occurrence of these species in Ovia River.

The relative spatial abundance among the analyzed zooplankton taxa was in the order of Station C > Station B > Station A. This was consistent for *Calanoida, Cyclopoida, Cladocera*, and *Harpacticoida*; except for *Amphipoda* which was in the order of Station B > Station C > Station A.

## Conclusion

The adults of the population are liable to elicit carcinogenic health challenges associated with lead and iron ingestion. The males are at risk of non-carcinogenic illnesses which have been linked to the combined toxicity effects of all the metals analyzed.

Iron, lead, TPH, and OG had a substantial impact on the general abundance of zooplankton in the aquatic habitat. The dominance of Cyclopoida in the aquatic habitat validates the significance of the pollution and confers bioindicator reputation on the zooplankton species for future biomonitoring studies. The pollution in Egbokodo River warrants a prompt remediation measure.

## Ethical statement

No human subjects were involved in this study. Standard reference data were adopted and estimations were conducted according to scientific standards.
